# Patient and public involvement and engagement in critical care research in low and middle-income countries: challenges and solutions

**DOI:** 10.62675/2965-2774.20250089

**Published:** 2025-09-11

**Authors:** Arishay Hussaini, Nikhat Ahmed, Maham Jawaid Ahmed, Madiha Hashmi, Timo Tolppa

**Affiliations:** 1 Ziauddin University Critical Care Research Group Karachi Sindh Pakistan Critical Care Research Group, Ziauddin University - Karachi, Sindh, Pakistan.

Better three hours too soon than a minute too late…

William Shakespeare

## INTRODUCTION

Critical care research holds immense potential to improve patient outcomes. However, involving critically ill patients in research is particularly challenging due to the unpredictable nature of acute illness.^(1)^ Patients often face high mortality risks and lack the capacity for informed decision-making, while their surrogate decision-makers experience severe emotional distress.^(1)^ In low- and middle-income countries (LMICs) like Pakistan, barriers such as low literacy rates, language differences, and limited public awareness about research further complicate involvement.^(2,3)^

A key strategy for making research more accessible for patients is to build partnerships with them and their families, for example, by establishing patient and public involvement and engagement (PPIE) groups.^(4)^ Such groups enable researchers to incorporate public perspectives in study design, conduct, and dissemination, enhancing research questions’ relevance, improving study materials’ clarity, and increasing community trust, leading to better recruitment, retention, and more ethical study processes.^(5-7)^

**Figure 1:**
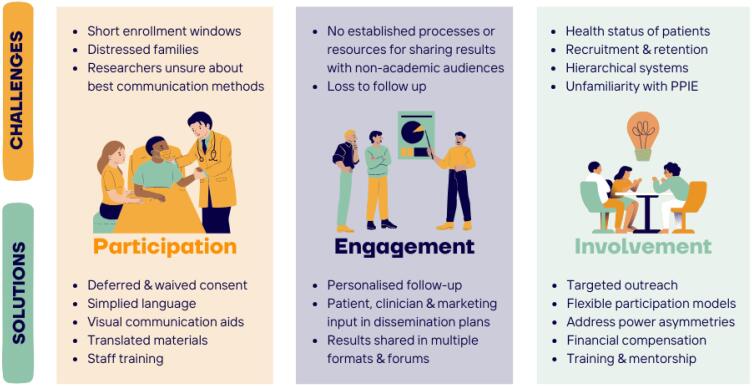
Challenges faced and their solutions.

We established Pakistan's first-ever PPIE group for critical care trials through collaboration between carers of critically ill patients and community representatives. Members were recruited through outreach in clinical settings and received basic training in research ethics.^(8)^ This article outlines key challenges and strategies across three levels of PPIE: participation, engagement, and involvement, as defined by the National Institute for Health and Care Research (NIHR), with each level reflecting a higher level of involvement and influence by the public.^(9)^ These lessons were also presented through a lecture to Brazilian researchers to support patient-centered strategies, especially in LMICs.(Figure 1).

PPIE - patient and public involvement and engagement

### Participation: patient-centered recruitment and consent discussions

The foundational level of PPIE involves patients taking part in studies as participants.^(9)^ Recruitment for critical care research is complex due to short enrollment windows that leave little time to contact and discuss studies with families.^(10)^ In Pakistan, family-based decision-making further complicates timely recruitment as decisions depend on the presence of key family members. Distressed families are often unwilling to engage with lengthy and technical consent forms. At the same time, study coordinators struggle to explain key research concepts. They are unsure about the right time to approach families, as Cezar et al. in this journal noted.^(10,11)^ Our group is exploring the acceptability of alternative consent models (i.e., deferred or waived consent) and research practices preferred by patients, as the evidence base in this area is limited.^(12)^

Through co-designing consent materials and processes and summarizing key information with patients, we have found that simplified language, visual aids (e.g., infographics and animations), and local language translations enhance understanding. Patients have expressed interest in materials that clearly outline risks and benefits, and for ongoing dialogue throughout the research process rather than a one-off consent discussion.

Staff face difficulties explaining research to families due to a lack of experience and challenges with translation. Training staff to communicate study information in culturally appropriate ways has improved participant understanding.^(10)^ We have also found that providing staff and families consent materials in local languages, such as Urdu, Punjabi, Pashto, Sindhi, and Balochi, helps. However, most languages do not have equivalent words for research terminology; if they do, the concepts do not have meaning or are not commonly understood.^(8)^ Researchers must be sensitive to this and produce clear, simple, and locally relevant study materials with patient input to promote patient-centered consent discussions.

### Engagement: communicating trial results and maintaining transparency

Engagement involves sharing information and disseminating research findings.^(9)^ Communicating trial results to participants remains challenging, particularly in intensive care units (ICU) settings where patients may have been unconscious or sedated during enrollment. Lack of post-study communication leaves participants and clinicians uninformed about trial outcomes.^(13)^ Logistical challenges, such as patient discharge or mortality before study completion, make it difficult to follow up with study participants. To address this, we are exploring strategies to improve follow-up processes, including phone calls, emails, and personalized letters that cater to patients’ preferences and health literacy levels. Creating simplified summaries using infographics and animated videos with marketing experts and organizing community forums will promote more effective dissemination. Engagement also requires ensuring findings are relevant to participants, families, and the broader community. Unfortunately, dissemination is often limited to academic audiences through papers and conferences. Patient and non-academic clinician feedback loops in dissemination strategies help create a sense of ownership and ensure research findings are relevant beyond the academic community.

### Involvement: equitable partnerships with patients

The ‘highest’ level of engagement involves integrating patient voices throughout the research process, from design to dissemination, in a collaborative partnership.^(9)^ However, meaningful involvement in critical care research can be difficult due to logistical, social, and financial constraints. Patients in ICU settings are often too unwell to participate during their hospital stay, and fluctuating health post-discharge can affect their long-term engagement. A significant difficulty lies in identifying individuals predisposed to developing critical illness, as it is difficult to predict who will require ICU admission, making the potential recruitment pool vast and undefined. Additionally, there is no routine follow-up system for ICU survivors in most LMICs, making re-engagement difficult. Our strategy focuses on post-discharge recruitment using structured follow-up strategies and targeted outreach. Flexible participation models, such as virtual meetings and asynchronous surveys, allow participants to engage at their convenience.

Addressing structural barriers to participation is also critical. In LMICs, hierarchies and patriarchal norms can limit certain voices in research discussions.^(14)^ To challenge such systems, we adopted an approach that avoids using formal titles, encourages majority representation of women in conversations, and employs strong facilitation to encourage open dialogue. Financial compensation for time and travel removes economic barriers and promotes equitable participation.

New members often face uncertainty and hesitation due to their unfamiliarity with PPIE. To build confidence and ensure active participation, we provide comprehensive training sessions through workshops covering research processes, healthcare systems, and PPIE objectives. Experienced members offer mentorship, fostering a supportive environment that encourages engagement. Additionally, a well-defined document outlining roles and responsibilities ensures participants understand their involvement and remain engaged.

Comparable efforts in other LMICs reinforce this approach. In Tanzania, village meetings were used to review findings and discuss interventions.^(15)^ In Nigeria, a task force with community representatives recommended culturally appropriate adaptations to stroke prevention studies.^(16)^

As PPIE becomes a funding requirement, risks of tokenism increase, and the involvement is symbolic, not substantive. We set realistic goals, co-develop outputs, enable shared decisions, and include our public partners as co-authors in academic activities to facilitate genuine involvement.

Our group relied on a modest Mahidol Oxford Tropical Medicine Research Unit bursary. However, true involvement remains challenging without sustainable funding. For PPIE to succeed, funders must treat it as core infrastructure and support coordination, training, and compensation.

Involvement fosters a sense of ownership and empowerment among participants, supporting the broader goals of equity and justice in global health research. Despite the complexities of critical care research in low—and middle-income countries, meaningful patient and public involvement and engagement are achievable and necessary. Patients, families, and public contributors are eager to engage when provided with appropriate opportunities to support. However, successful involvement requires active efforts to address barriers, challenge cultural and systemic inequities, and establish sustainable participation models.

### Key message

Patient and public involvement and engagement promote the relevance, accessibility, and acceptability of critical care research and are achievable in low—and middle-income countries, such as Pakistan.
